# Case of Strangulated Inguinal Hernia, Reduced on the New Method Recommended by Dr. Andrew Buchann, Professor of Institutes of Medicine in the University of Glasgow

**Published:** 1847-11

**Authors:** Archibald Wallis Mackie

**Affiliations:** Cupar, Fife


*Case of Strangulated Inguinal Hernia, reduced on the New Method recommended by Dr. Andrew Buchann, Professor of Institutes of Medicine in the University of Glasgow. By* ARCHIBALD WALLIS MACKIE, *Cupar, Fife.*—G. M., aged seventeen years, railway labourer, of a stout habit of body, and enjoying previous good health, whilst employed lifting some heavy railway sleepers on Friday last, felt something to give way at the lower part of his abdomen. The patient was unable to walk, and was carried to a neighbouring house, where he remained till next day, when he was conveyed to his father's residence, a distance of eleven miles. I was called to visit him on Sunday morning, and on examination found a tumour the size of a hen's egg, situated in the right iliac region, the general characters of which led me to conclude that it was a case of strangulated oblique inguinal hernia. The patient had not had his bowels opened since the morning of the accident. I ordered him an enema, and, after waiting till it was expelled, I applied the taxis, but unsuccessfully; I then had recourse to the usual remedies adopted in such cases, but without any effect. I bethought me of the plan recommended by the talented Professor of Physiology in the Glasgow University, and I was glad to see my efforts crowned with success. The mode is very simple. I placed my patient on his back, flexing the thighs on the pelvis, and putting the muscles of the abdomen in as relaxed a condition as possible. I then desired the patient to empty his lungs of as much air as possible, and having an assistant at hand, who immediately held his nose and mouth to prevent inspiration, I applied gentle pressure over the tumour in the proper direction, and had the satisfaction to feel it give way, and, as it were, *draw up* into its natural cavity.

The *rationale* seems to me to be, when the lungs are emptied of air, the diaphragm is, as it were, sucked up to fill the diminished thoracic cavity; it (diaphragm) exerts a tractile power over the floating viscera of the abdomen, and draws the protruded intestine upwards—naturally *assisting*, if not altogether accomplishing the reduction of the hernia.

Such is the mode, I conceive, in which the reduction is accomplished; and I have no doubt that, in addition to the mechanical influence, the temporary suspension of the breathing must have a powerful sedative effect, and consequently a relaxing influence, on any part morbidly constricted. Before operating I would always give this plan a fair and impartial trial, and I am confident, if practitioners would adopt this method, they would have the satisfaction of relieving their patients, and thus averting the dangers of a painful and often fatal operation.—*Ibid.*

